# A Broadband Bessel Beam Launcher Using Metamaterial Lens

**DOI:** 10.1038/srep11732

**Published:** 2015-06-30

**Authors:** Mei Qing Qi, Wen Xuan Tang, Tie Jun Cui

**Affiliations:** 1 State Key Laboratory of Millimeter Waves, Southeast University, Nanjing 210096, China; 2 Synergetic Innovation Center of Wireless Communication Technology, Southeast University, Nanjing 210096, China

## Abstract

An approach of generating broadband Bessel beams is presented. The broadband Bessel beams are produced by a gradient index (GRIN) metamaterial lens illuminated by broadband waveguide antenna. The metamaterial lens is constructed with multi-layered structure and each layer is composed of GRIN metamaterials. The metamaterials are designed as dielectric plates printed with metallic patterns in the center region and drilled by air holes near the edge, which operate in wide band. The metamaterial lens serves as a convertor which transforms the spherical beams emitted from feed into conical beams. The conical beams form quasi-Bessel beams in the near-field region. The aperture diameter of the GRIN lens is much larger than the operating wavelength to guarantee the transformation. In principle, this kind of metamaterial lens can produce Bessel beams at arbitrary distance by designing the refractive-index distribution. To verify the approach, we have designed, fabricated and tested a metamaterial lens. Full-wave simulation and experiment results have proved that the generated Bessel beams can be maintained in distance larger than 1 meter within a ranging from 12 GHz to 18 GHz.

The non-diffracting beam solutions to the free-space homogeneous Maxwell’s equations, i.e., the Bessel beams, were proposed by Durnin in 1987^1^. The beam exhibits the property of remaining confined and not suffering diffractive propagating. Bessel beams are of considerable potential applications such as electromagnetic propulsion, remote power transmission, and secure communication. An ideal Bessel beam can be thought to be a superposition of plane waves with wave vectors lying on a cone. This is the basis idea for launching Bessel beams. However, the truth is that an ideal Bessel beam does not exist due to the fact that they are not square integrable. In practice, a truncated Bessel beam, termed as the quasi-Bessel beam, can be realized with the diffraction-free characteristic of remaining main lobe up to a significant distance^2^.

In the latest decades, there have been a series of studies on the method of launching Bessel beams. In optics, the first experiment for generating Bessel beam was presented by Durnin^3^. Later, an axicon lens as a Bessel beam launcher was proposed by Herman and Wiggins, and rapidly became widely-used approach for forming Bessel beams in optical experiment^4^. Holograph illuminated by a laser beam is another proposal to generate Bessel beam. Its experimental arrangement was based on a holographic optical element to generate Bessel beams efficiently^5^,^6^. Pendry presented the method of localized waves to launch Bessel beams^7^. Recently, to help the user to avoid preliminary optical adjustments, axicon on a gradient index lens for Bessel beam generation from a point-like source was proposed by Xie^8^.

To be remarked, it is more difficult to achieve a plane wave in a small distance to illuminate the axicon lens at microwave frequency than in optics. In fact, in the microwave or millimeter (mm)-wave regime, the implementation of Bessel beams is much less reported. The first generation of Bessel beams in the millimeter-wave region of the spectrum was proposed by Monk *et al.* in 1999 using an axicon^9^. The microwave launcher of Bessel beams was based on the study by Mugnai in 2000^10^. In 2001, a computer-generated binary amplitude hologram was used to transform an initial Gaussian electromagnetic field with spherical phase front into a non-diffracting Bessel beam^11^. By locating a circular grating-like structure in front of a subwavelength aperture, a diffraction-free beam can also be generated in microwave region^12^. A new method was presented by Salem *et al.* that a truncated Bessel beam was set to be the aperture field at the open-end of a flanged metallic circular waveguide section^13^. By controlling the excitation currents of the loop antennas in the waveguide, one was able to manipulate the beam intensity, spot size and modulation. Recently, Grbic and coworkers presented several new approaches for generating Bessel beams in microwave regime, including leaky-wave modes^14^, near-field plates for evanescent Bessel beams^15^, and metamaterial Huygens’ surfaces^16^,^17^. Caloz and coworkers presented a method of generating Bessel beams using two-dimensional antenna array at millimeter-waves^2^. The method based on a radial line slot array was also proposed at millimeter-wave frequency to generate Bessel beams^18^.

More recently, Cui *et al.* proposed a method to realize planar Bessel beams using artificial meta-surfaces^19^. This design benefited from the metamaterial that has the ability to manipulate electromagnetic waves flexibly. Metamaterials are artificial materials composed of periodic structures in the sub-wavelength scales, and have provided an unprecedented skill to control electromagnetic waves and create new devices such as invisibility cloaks^20^,^21^,^22^,^23^,^24^, perfect absorbers^25^,^26^, metamaterial lenses^27^,^28^,^29^,^30^,^31^ and metamaterial antennas^32^,^33^,^34^,^35^. Among various metamaterials in literatures, gradient refraction index (GRIN) metamaterials which possess gradient permittivity and/or permeability have played an important role. The GRIN metamaterials can be artificially implemented with different unit cells with varying geometries and dimensions, and therefore, is simple to design. Since the first GRIN metamaterial^27^ the GRIN metamaterials have been utilized in achieving a variety of microwave applications, such as Luneburg lens^30^,^31^, highly directive antennas^32^, Maxwell fish-eye lens^33^,^34^, low sidelobe lens antennas^35^, and beam-scanning antennas^29^.

In this work, we present a novel method to generate the Bessel beams in a wide bandwidth using broadband gradient index (GRIN) metamaterial lens. Through manipulating the distribution of refraction index, the GRIN lens transforms spherical waves into conical waves and forms a Bessel beam within a distance named as the maximum propagating distance (Z_max_), which can be tuned by changing the refraction index distribution. It is remarkable that, up to date, only a few of the proposed approaches for launching Bessel beams can operate in a wide bandwidth. However, proposed GRIN lens works in a very wide band because the transformation of wave fronts are frequency-independent, and the metamaterial unit cells adopted to construct the lens preserve the same refraction properties in the wide band. Full-wave numerical simulation and measurement results validate the predictions.

## Results

### Mechanism of the Bessel beam launcher

As shown in Ref. 4, an axicon can be adopted as the Bessel beam launcher. In fact, such an axicon is a converter which transforms plane waves into conical waves. In microwave, however, an ideal plane wave is hard to generate. Moreover, the reflection at the interface between the axicon and the air also decreases the efficiency. To implement a simple, low-loss, and operating in broadband Bessel beam launcher, a novel method of Bessel beam launcher based on GRIN metamaterial lens is proposed here.

**Figure 1:**
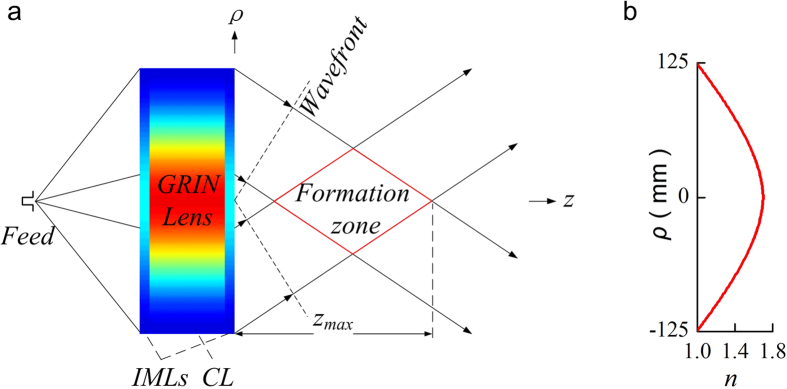
Schematic diagram and refraction index distribution of the broadband Bessel beam launcher. (**a**) Schematic diagram. (**b**) Refraction index distribution along radial direction.

It has been shown that a well-designed planar GRIN metamaterial lens can transform a point source with spherical wavefront to a deflection beam^29^. If we make the refraction index to have a special axially-symmetric distribution, a conical wavefront will be directly generated, as illustrated in Fig. 1(a). This Bessel beam launcher includes two parts: one is the planar GRIN metamaterial lens and the other is a feed with axially symmetric radiation patterns^36^. In contrast to the axicon setup which needs Gaussian beams for excitation, the GRIN lens is illuminated by a nearly spherical wave emitted from the feed, a broadband waveguide antenna. The GRIN metamaterial lens is designed to transform the spherical beam to the conical wave. The formation zone shown in the figure is the area of existing Bessel beams. Therefore, in other words, the GRIN lens here is a transformer that converts a spherical wave to a Bessel beam. To be noted, to reduce the reflection loss, two impedance matching layers (IMLs) are used to cover the core layer (CL) of the GRIN lens^35^. Aditionally, the paraxial approxiamtion is assumed in the design, therefore, the launcher is designed based on the refractive index of the lens without consideration for polarization conversion. The polarization of the beam is based on that of the feed^36^.

Moreover, we can change the formation zone through adjusting the deflection angle of the beam, and the deflection angle is determined by the refraction index of the GRIN lens. Therefore, we can manipulate the Bessel beams through designing refraction index of the lens. The relative phase distribution of the aperture field is designed to have the following form as:


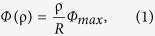


in which *R* is the lens’ radius, and *Φ*_*max*_ is the relative phase of electric field at the edge which is determined by the refraction index of the lens. According to the geometrical relation shown in Fig. 1, the maximum propagating distance of the Bessel beam *Z*_*max*_ can be obtained as


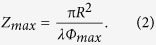


From Eq. (2), we conclude that the maximum propagating distance of the Bessel beam is determined by three factors: *R*, *λ*, and *Φ*_*max*_. The larger *R* is, the larger *Z*_*max*_ is; the smaller *λ* or *Φ*_*max*_ is, the larger *Z*_*max*._ is. Therefore, we can obtain Bessel beams in a longer distance through reducing *Φ*_*max*_ when the other two factors are fixed. Moreover, when the product of *λΦ*_*max*_ is fixed, the range of the Bessel beam is a constant and does not change with operating frequency. That is, in this case, the launcher is operating in a broad band.

### Design process of the Bessel beam launcher

We designed a GRIN metamaterial lens operating in the whole Ku band to validate the approach above. To obtain the phase distribution illustrated in Eq. (1), a gradient refraction index distribution is designed as follow:


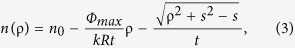


where, *n*_*0*_ is the refraction index at *ρ* = 0, *k* is the free space propagation constant, and *s* is the distance of the feed away from the GRIN lens. The lens’ radius *R* = 125 mm and thickness *t* = 100 mm are selected. The thickness of each IML is 4 mm. The rectangular waveguide antenna is adopted as the feed and placed *s*  = 78 mm away from the GRIN lens. The *Φ*_*max*_ is selected as 2π/3 at the center frequency of 15 GHz. From Eq. (3), we know that the refraction index decreases as *ρ* increases. Therefore, the minimum refraction index will emerge at the edge of the lens. To guarantee the minimum refraction index larger than one in practice, *n*_*0*_ is selected as 1.7. The theoretical value of *Z*_*max*_ is 1.2 m at the center frequency. The distribution of the refraction index is shown in Fig. 1(b). The refraction index ranges from 1.0 to 1.7. The index can be easily realized through drilled-hole dielectrics or metallic-ring particles.

**Figure 2:**
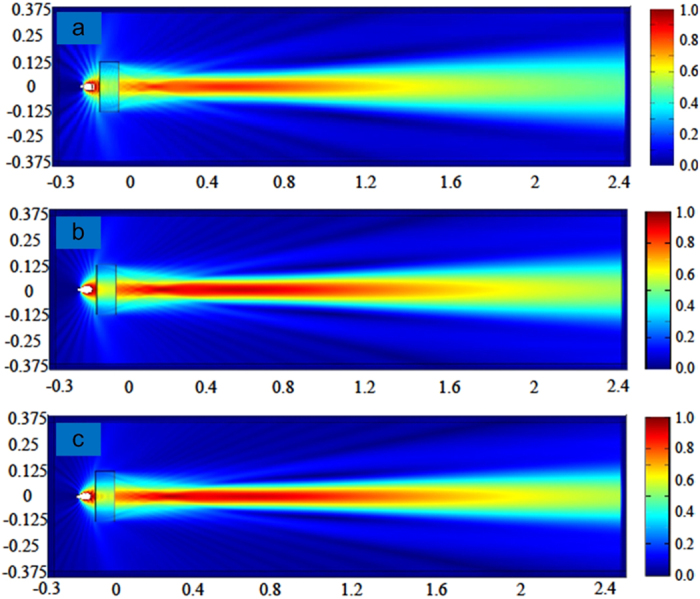
Electric field distribution of 2-D Bessel beams generated by the lens at different frequencies. (**a**) 12 GHz; (**b**) 15 GHz; (**c**) 18 GHz. (dimension unit: m).

We computed the property of the device in the simulation tool of COMSOL. The boundary is set to be perfectly absorbing boundary. The point source is placed in an open-ended waveguide to produce TE mode wave. The calculated field is obtained within the distance of 2.4 m away from the lens. The relative electric field generated by the GRIN lens at three sample frequencies, 12, 15 and 18 GHz, are shown in Fig. 2, respectively. It can be observed clearly that the beams generated by the lens maintain in a distance more than 1 m non-diffractive at these frequencies. Moreover, the non-diffraction range is longer at higher frequency. This agrees well with Eq. (2) that *Z*_*max*_ increases as *λ* decreases.

**Figure 3:**
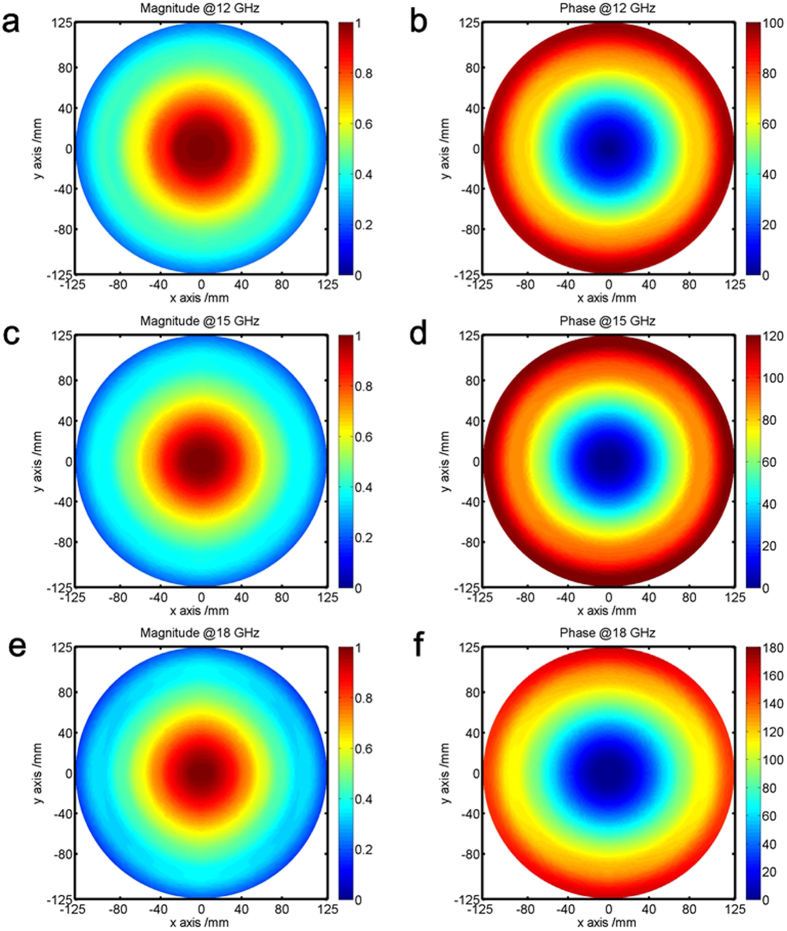
Electric field distributions at the aperture of the proposed GRIN lens at different frequencies. (**a**,**b**) 12 GHz. (**c**,**d**) 15 GHz. (**e**,**f**) 18 GHz.

To further investigate the property, we have also performed the numerical simulations of three-dimension (3D) metamaterial lens using CST Microwave studio. The GRIN lens is constructed with a series of concentric annulus. Each annulus is made of one kind of dielectric with permittivity corresponding to the designed refraction index distribution. The transverse component of electric field distributions at the aperture of the GRIN lens, including amplitude and phase, are depicted in Fig. 3. It can be observed that the distributions are rotationally symmetric, which is guaranteed by the feed with axially symmetric radiation patterns^36^. The symmetric property of the aperture field also guarantees the symmetric property of the beams generated by the lens. The amplitude distributions of the field are tapered ones along the radial direction, which to some extent mimics the Gaussian function. The phase distributions along the radial direction are linear, which is in good agreement with the relation shown in Eq. (1). The maximum relative phase at 15 GHz has a value of *Φ*_*max*_ = 2π/3.

**Figure 4:**
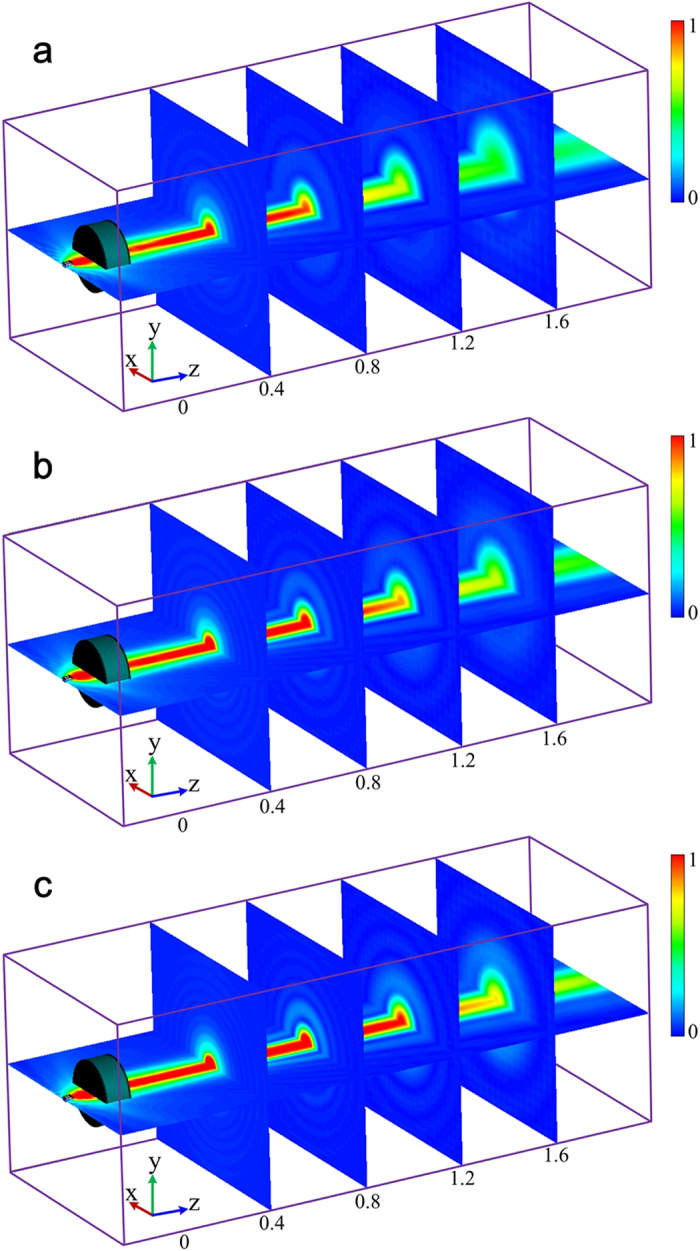
Electric field distributions of 3-D Bessel beams generated by the lens at different frequencies. (**a**) 12 GHz; (**b**) 15 GHz; (**c**) 18 GHz. (dimension unit: m)

**Figure 5:**
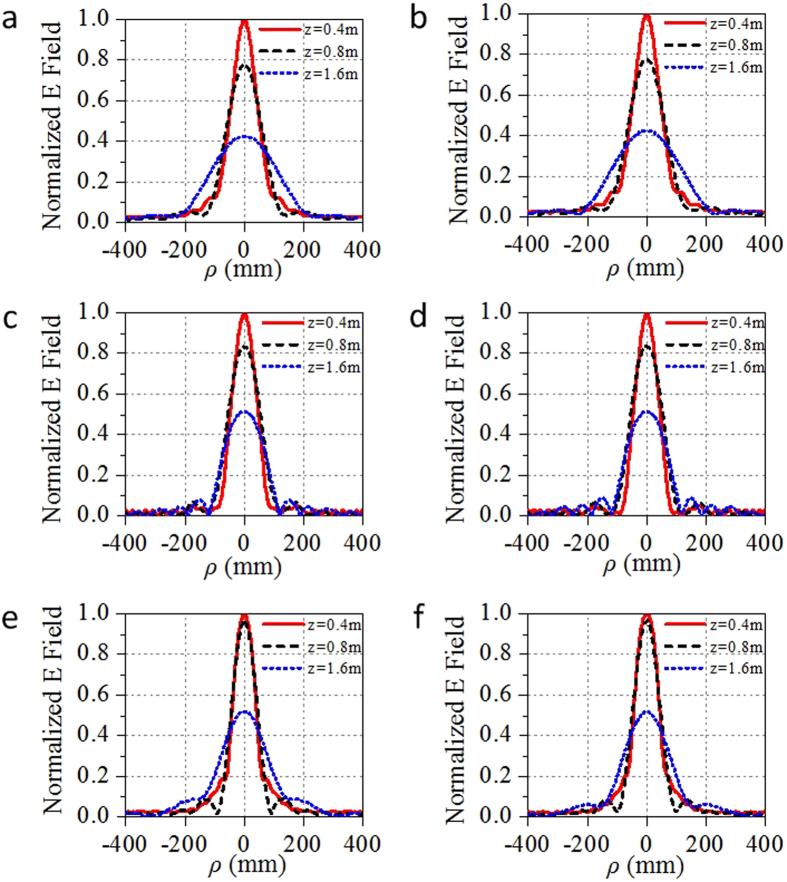
Transverse profiles along E- and H-direction at various distances from the lens. (**a**) In YOZ plane at 12 GHz. (**b**) In XOZ plane at 12 GHz. (**c**) In YOZ plane at 15 GHz. (**d**) In XOZ plane at 15 GHz. (**e**) In YOZ plane at 18 GHz. (**f**) In XOZ plane at 18 GHz.

**Figure 6:**
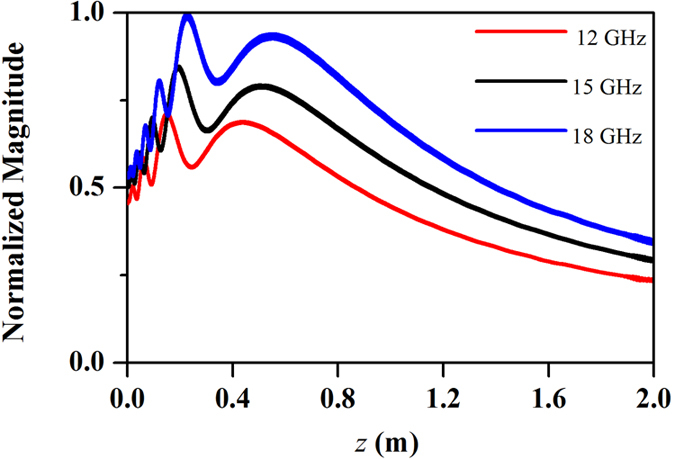
Normalized Magnitude of E field along z-axis at 12, 15 and 18 GHz, respectively.

The beams generated by the GRIN lens at three sample frequencies, 12 GHz, 15 GHz and 18 GHz, are shown in Fig. 4. The beams propagate along the longitudinal direction (along z-axis here) without diffraction in a distance more than 1 m. Moreover, the propagating distance at higher frequency is longer than that at lower frequencies. The transverse component of electric field distributions in the transverse planes *z* = 0.4, 0.8, 1.2, and 1.6 m are also shown in Fig. 4. We notice that the beams are confined in a small region (about several wavelengths) and are axially symmetric. Especially, in the region from *z* = 0.4 m to 1.2 m, the beams are emerged as good diffraction-free beams. The energy of the beams is gathered near the longitudinal axis for a long distance, and the beam width in the transverse direction is about several wavelengths. To further quantify the non-diffractive properties of generated beams, the profiles of the beam energy along transversal directions at different position of the optical axis are shown in Fig. 5. The energies for the beams are confined in a beamwidth narrower than 0.1 m as the sidelobes of the beams are significantly low with respect to the main lobe. Moreover, the shapes of the beams are almost the same at different frequencies. Therefore, this Bessel beam launcher has the ability of operating in a broad band. The profiles of the beam energy along the optical axis are shown in Fig. 6. The energies are significantly fluctuant for a distance near the lens and then maintain a high level for a distance before they decay. This is the common phenomenon for the Gaussian-Bessel beam.

### Implementation of the Metamaterial Lens

It should be noted that the GRIN lens is modeled in CST microwave studio using a series of cylinder dielectrics with the correlated designed permittivities. In practice, dielectrics with different permittivities are hard to be realized directly. To implement the GRIN lens, the theory of equivalent medium is used, that is, the metamaterial unit cells are used to replace the ideal dielectrics.

**Figure 7:**
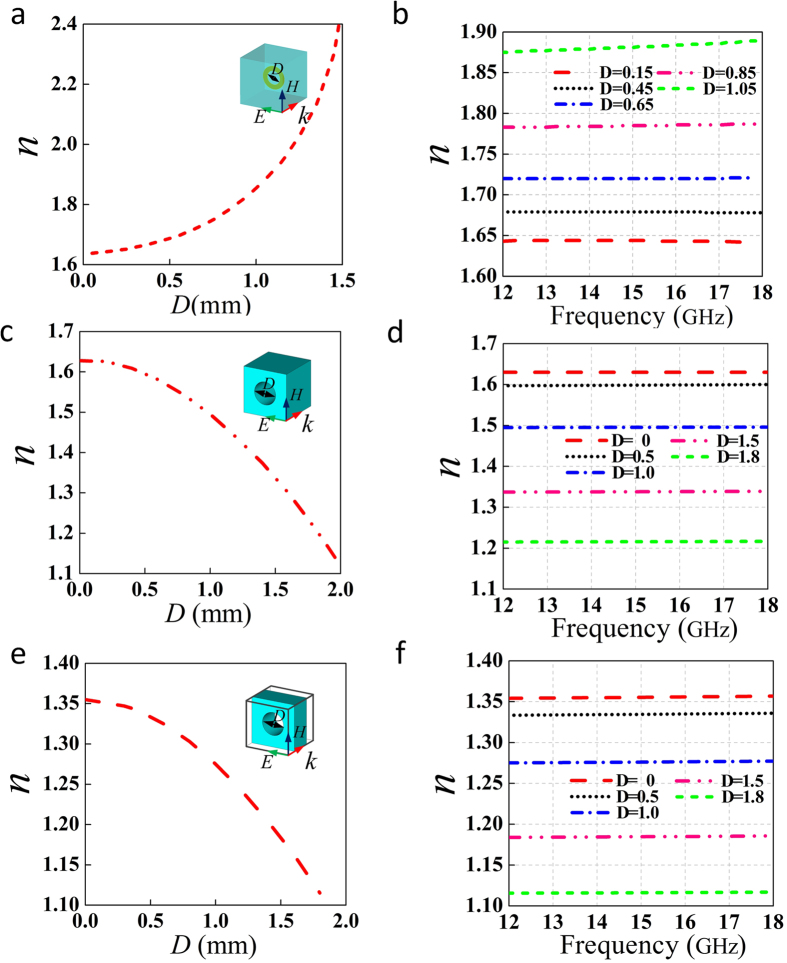
Characteristics of the metamaterial unit cells. (**a**,**b**) Metallic ring. (**c**,**d**) Drilled-hole unit cell with thickness of 2 mm. (**e**,**f**) Drilled-hole unit cell with thickness of 1 mm.

To guarantee the launcher to be broad band, the GRIN lens should maintain the same refraction index distribution in the whole operating band. Therefore, metamaterial unit cells adopted here should have broadband property as well. Three kinds of metamaterial unit cells have been used to construct the GRIN lens. The first one is metallic rings printed on F4B substrate with relative permittivity 2.65 and loss tangent 0.001. The second one is F4B unit cells with drilled holes. The thickness of the F4B substrate is 2 mm. The third one is similar to the second one but the thickness of the F4B substrate is 1 mm. The properties of these unit cells are shown in Fig. 7. For the metallic ring, the refraction index decreases as the diameter of the rings (*D*) decreases. The refraction index is larger than that of the substrate. For the drilled-hole substrate, the refraction index decreases as the hole diameter (*D*) increases. For the unit cell with thinner substrate, the smaller refraction index can be achieved. The relation between the refraction index and the frequency are also shown in Fig. 7 for the three kinds of unit cells. For the metallic-ring unit cells, the small ring can maintain the same refraction index across the Ku band. But for the large ring (for example, *D* = 1.05 mm), the refraction index will slightly increases as the frequency increases. This is because that the resonant frequency is lower for the larger ring and it approaches the operating frequency. Note that the maximum refraction index for designed lens is 1.7, and the largest ring adopted in the lens has *D* = 0.97 mm. Therefore, the metallic rings adopted in the lens can maintain constant in the operating frequency band. For the drilled-hole F4B unit cells, the refraction index maintains almost the same when the dimension of *D* varies. To conclude, the refraction index of all metamaterial unit cells used in the lens remains almost constant within the entire Ku band and they are all able to operate in a broad band.

To be noted, the required refraction index in the center region of the lens is larger than the index of F4B. So the metallic-ring unit cell is designed to satisfy the larger index. The drilled-hole unit cells are used to satisfy the refraction index smaller than that of F4B.

## Discussion

**Figure 8:**
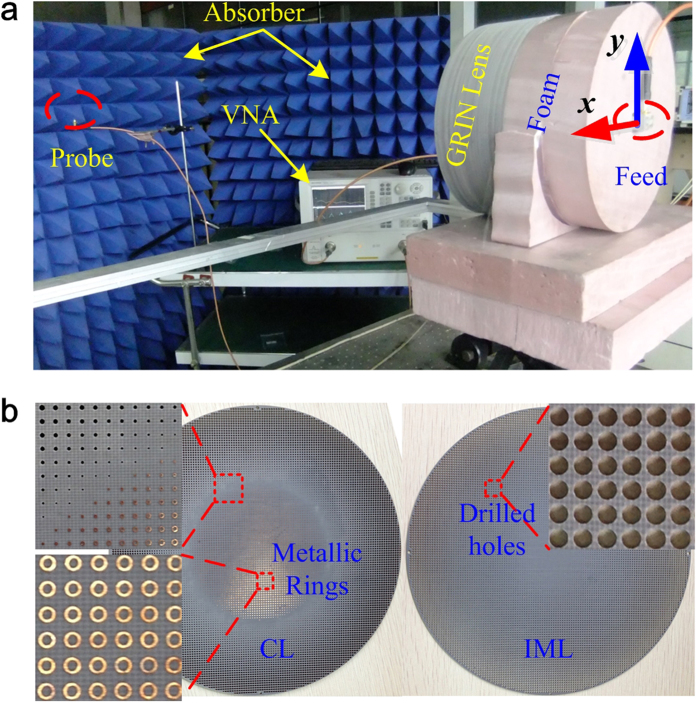
Photos of the Bessel beam launcher and the experimental setup. (**a**) The experimental setup. (**b**) Details of the metamaterial plates.

We finally constructed the broadband Bessel beam launcher shown in Fig. 8. The GRIN metamaterial lens consists of multilayer plates. The substrate is F4B. For the core layer of the lens, because the required refraction index is larger than that of F4B when the radius is smaller than 60 mm, metallic rings with different scales are printed on one side of the plate in the center region. The diameter of the ring decreases as the radius increases. For the region where the radius is larger than 60 mm, the substrates are drilled with air holes. The diameter of the hole increases as the radius increases. The thickness of each plate is 2 mm, and 46 pieces of the same plates all together form the core layer of the lens. For the impedance matching layer of the lens, the plates are all drilled with air holes. The thickness of each plate is 1 mm and 4 pieces of the same plates construct the IMLs. To ne noted, there is 1-mm-thick foam plate added between neighboring F4B plates.

The setup for measuring the electric field is also shown in Fig. 8. The lens is fed by a broadband waveguide antenna placed 78 mm away from the lens. A foam block with relative permittivity about 1.05 was adopted to separate the feed and the GRIN lens. The feed is a special open-ended waveguide antenna which possesses axial symmetric radiation patterns^36^. The electric field emitted from the feed is mainly in y direction. So the *E*_*y*_ component is the main electric field for the generated beams. The Bessel beam launcher is placed on a 2D stepping platform. The electric probe used was approximately 2 mm which is one tenth wavelength at the center frequency. The probe used for detecting the transverse component of electric field is fixed at different positions away from the lens. We used Agilent vector network analyzer to test the signal. The launcher and the probe were enclosed by radar absorbing materials to reduce the spurious interference.

**Figure 9:**
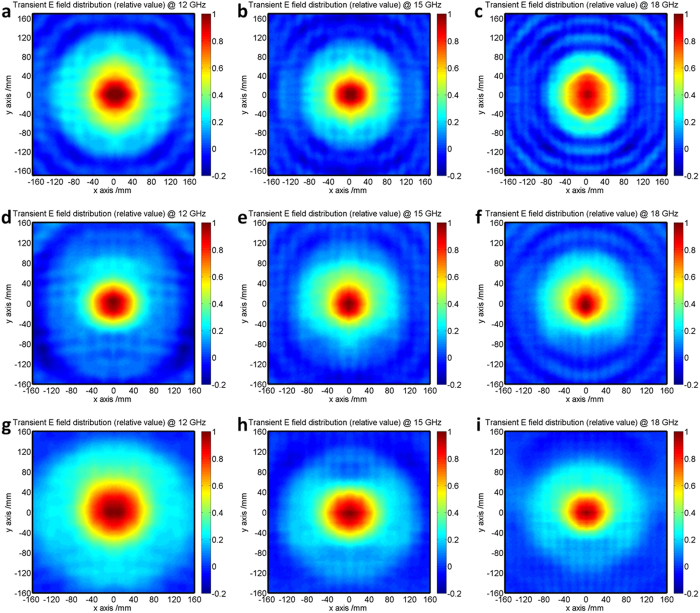
Experimental transversal profiles at different transversal planes at 12, 15 and 18 GHz, respectively. (**a**–**c**) z = 0.4 m. (**d**–**f**) z = 0.6 m. (**g**–**i**) z = 0.8 m.

Figure 9 illustrates the experimental transversal profiles of the beams at *z* = 0.4, 0.6 and 0.8 m planes. There exists a main spot in the center of each figure. The spot is the main lobe of the Bessel beams. The width for the spot is around 80 mm. That is, the width of the generated Bessel beam is about 4 times of the wavelength at the center frequency. If we increase the transverse wavenumber of the beam, a narrower beam width can be obtained. Of course, the propagating distance for the existing Bessel beams will decrease accordingly. The field distributions are rotationally symmetric. Around the main spots, several very low side-lobes can be observed. This is in good agreement with the Bessel function.

**Figure 10:**
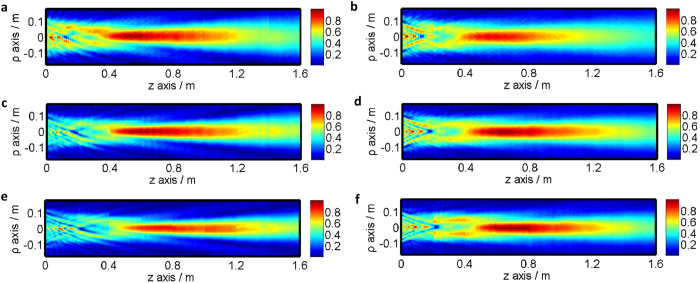
The measured E-field distribution in YOZ plane and XOZ plane. (**a**,**b**) 12 GHz. (**c**,**d**) 15 GHz. (**e**,**f**) 18 GHz.

The field profiles in H-plane (XOZ plane) and E-plane (YOZ plane) at 12 GHz, 15 GHz and 18 GHz are measured respectively in Fig. 10. It is observed that the energy is mainly confined around the optical axis and propagates along the axis over considerable distance (more than 1 m) without diffraction. The 3-dB beam widths for the beams are about 80 mm for both the H-plane and the E-plane. The beams for H-plane are specular symmetric. While the beams for E-plane are not specular near the lens aperture. This is due to the fact that although the GRIN lens is symmetric for both E-plane and H-plane, the feed is only symmetric for H-plane, because the rectangular waveguide is fed by a probe along the E-field direction.

In summary, we have proposed an approach to generate broadband Bessel beams with a long propagation distance using the GRIN metamaterial lens. The metamaterial lens transforms spherical beams to conical beams and constructs truncated Bessel beams within the formation zone. Numerical simulation and experiment results show that the Bessel beam can be preserved in a distance more than 1 meter in a broad band ranging from 12 GHz to 18 GHz. Metamaterials composed of dielectric plates printed with metallic patterns and dilled by air holes are implemented. It should be pointed out that there is a trade-off between the electrical thickness and the bandwidth of our design. If one only needs a non-diffracting beam operating in narrow band, a meta-surface with low profile can be adopted to replace the thick metamaterial lens.

## Methods

Numerical simulations of the Bessel beam launcher were performed by the commercial software, COMSOL and CST Microwave Studio. The material used to fabricate the feed was aluminum. The substrate printed with varies dimensions was F4B with the relative permittivity 2.65 and loss tangent 0.001. We used Agilent Vector Network Analyzer to measure the electric field of the fabricated Bessel beam launcher through using a small probe.

## Additional Information

**How to cite this article**: Qing Qi, M. *et al.* A Broadband Bessel Beam Launcher Using Metamaterial Lens. *Sci. Rep.* **5**, 11732; doi: 10.1038/srep11732 (2015).
